# Trouble in Tiger country: isolated traumatic pancreatic transection – a case report

**DOI:** 10.1093/jscr/rjad071

**Published:** 2023-02-23

**Authors:** Ahmed Faidh Ramzee, Zeenat Khuda Bakhsh, Hisham Al Jogol, Sandro Rizoli, Ruben Peralta, Ayman El-Menyar, Hassan Al-Thani, Ammar AlMadani

**Affiliations:** Department of Surgery, Trauma Surgery, Hamad General Hospital, Hamad Medical Corporation, Doha, Qatar; Department of Surgery, Trauma Surgery, Hamad General Hospital, Hamad Medical Corporation, Doha, Qatar; Department of Surgery, Trauma Surgery, Hamad General Hospital, Hamad Medical Corporation, Doha, Qatar; Department of Surgery, Trauma Surgery, Hamad General Hospital, Hamad Medical Corporation, Doha, Qatar; Department of Surgery, Trauma Surgery, Hamad General Hospital, Hamad Medical Corporation, Doha, Qatar; Department of Surgery, Trauma Surgery, Clinical Research, Hamad General Hospital, Doha, Qatar; Department of Clinical Medicine, Weill Cornell Medical College, Doha, Qatar; Department of Surgery, Trauma Surgery, Hamad General Hospital, Hamad Medical Corporation, Doha, Qatar; Department of Surgery, Trauma Surgery, Hamad General Hospital, Hamad Medical Corporation, Doha, Qatar

## Abstract

Isolated pancreatic transection is a rare surgical condition that occurs more commonly following blunt abdominal trauma. It carries a high degree of morbidity and mortality, and the management remains a source of debate as universally accepted guidelines are not well established owing to the paucity in clinical experience and large series. We presented a case of an isolated pancreatic transection following blunt abdominal trauma. The surgical management of pancreatic transection has evolved over the decades from aggressive approaches to more conservative measures. Given the lack of large series and clinical experience, no universal consensus exists, except for applying damage control surgery and resuscitation principles in critically unstable patients. For transections of the main pancreatic duct, most recommend excision of the distal pancreas. Concerns over the iatrogenic complications of wide excisions, particularly diabetes mellitus, have led to reconsideration and more conservative approaches, but it may fail in some cases.

## INTRODUCTION

Pancreatic trauma is rare, with an incidence ranging from 0.2% of all traumas to 3–12% of abdominal traumas [1–5]. Furthermore, isolated blunt pancreatic trauma is infrequent event occurring in 0.7%, in which blunt traumatic pancreatic transection (BTPT) is limited to a few case reports [6]. The infrequency of such lesion is one of the reasons for the lack of widely accepted guidelines on the management of these injuries [2]. Moreover, irrespective of the management, BTPT is usually associated with a high degree of morbidity and mortality.

The pancreas is a retroperitoneal organ and BTPT occurs almost invariably with other intra-abdominal organ and vascular injuries, due to the significant degree of force required to transect the pancreas [6]. The management of BTPT has been debated for decades with earlier practitioners advocating for an aggressive surgical approach. In contrast, more recent reports advocate more conservative non-surgical strategies [1]. We present a rare case of an adult patient who had isolated grade IV pancreatic injury (complete transection), following blunt abdominal trauma.

## CASE PRESENTATION

**Figure 1:**
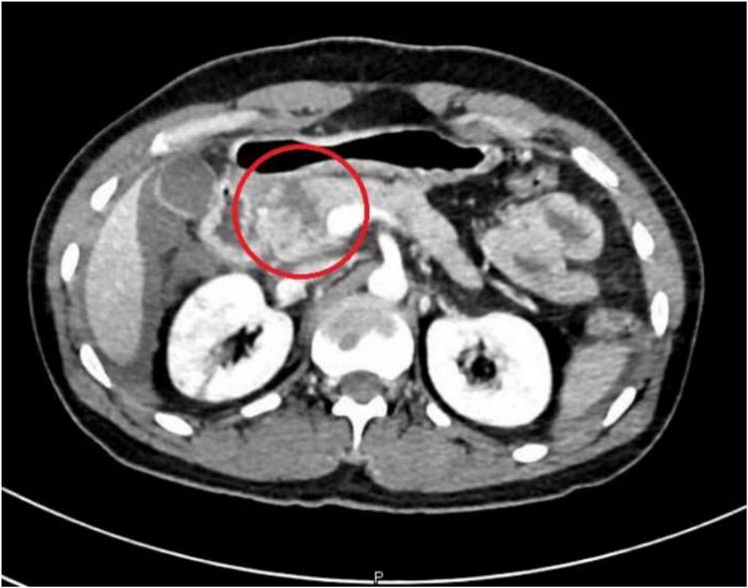
CT abdomen—sagittal section, showing transection of the pancreas (circle).

**Figure 2:**
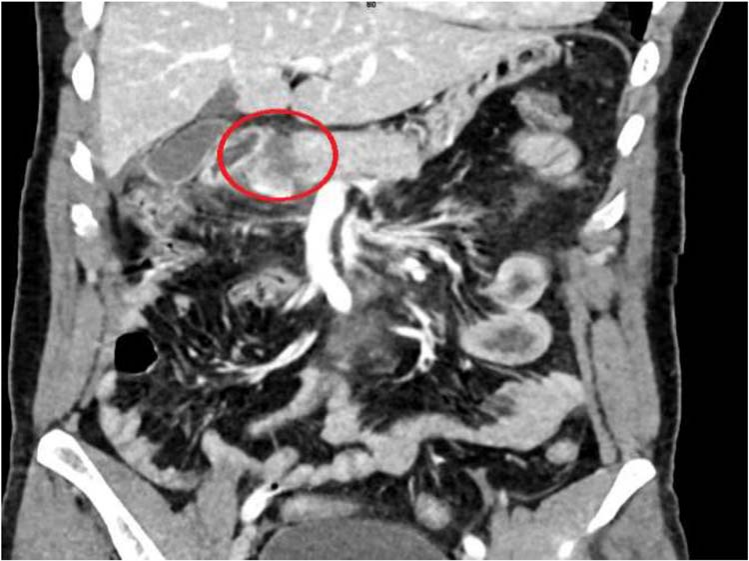
CT abdomen—coronal section, showing transection of the pancreas (circle).

A 36-year-old male manual laborer was assaulted by his roommates (kicked in the abdomen multiple times) and was intoxicated with alcohol. He was writhing in pain, hemodynamically stable with a guarding abdomen. Initial laboratory results showed high serum amylase level 485 U/L, high serum lipase 1200 U/L and elevated liver enzymes. A computed tomography (CT) scan revealed an enlarged bulky head of the pancreas with complete transection of the pancreatic neck (Figs 1–2). In addition, he had a small contusion of the liver and free fluid in the abdomen.

An exploratory laparotomy and pancreatectomy were planned. There was 500-ml fresh blood in the abdomen, a retroperitoneal hematoma along the duodenum and the root of the mesentery.

**Figure 3:**
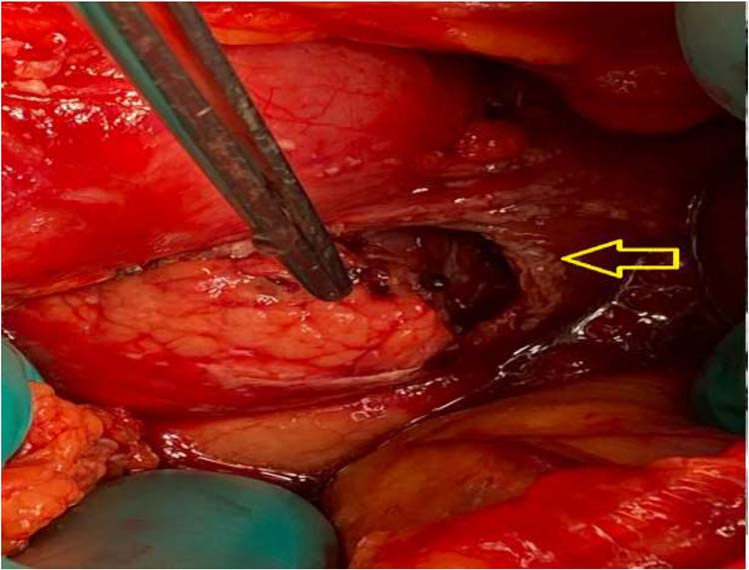
Intraoperative: pancreas seen through a laceration in the lesser sac (arrow).

**Figure 4:**
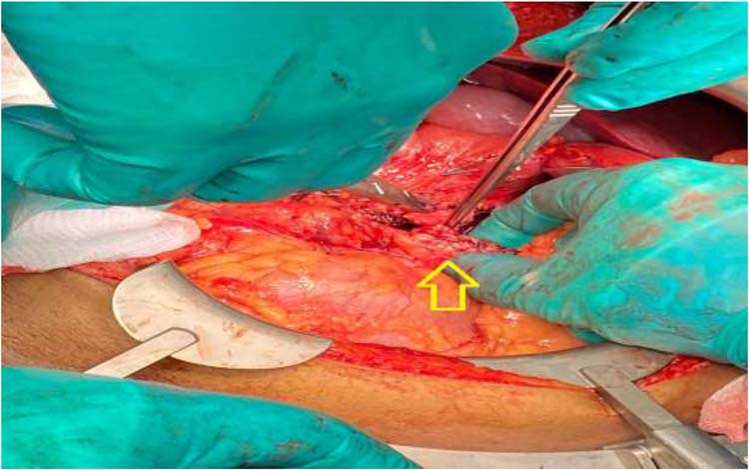
Intraoperative: transected ends of pancreas oversewn (arrow).

The pancreas was bulging through a rent in the lesser sac and completely transected just to the right of the superior mesenteric vessels. A 2-cm rim of the pancreatic head remained attached to the duodenum (Figs 3 and 4). There were no other lacerations, gross contusion or crushed portions of the pancreas.

Considering the location of the pancreatic transection, the small size of the remaining pancreatic head and the overall appearance of the pancreas, the intra-operative decision was modified. Instead, we opted not to excise the pancreas. The distal and proximal ends of the transected main pancreatic ducts were identified and directly sutured with 5.0 polypropylene sutures. The cut ends of the pancreas were oversewn with vicryl sutures and subsequently covered with the omentum.

The immediate post-operative period was uneventful; drain outputs averaged around 100 ml per day. The patient was doing well and was discharged home 2 weeks later with the drains in place with a weekly follow-up. However, he did not show up for a month. Later on, he attended the emergency department because the drains were unintentionally removed, and he was in pain. His CT scan revealed a peripancreatic collection measuring 88 mm × 17 mm × 52 mm, which was defined by the magnetic resonance cholangiopancreatography as a leak from a secondary branch of the main pancreatic duct of the distal pancreas, at the transected margin. Therefore, interventional radiology guided drainage of the collection was performed. Over the subsequent days, the drain output averaged 150 ml/day. However, due to the persistent pancreatic fistula, a distal pancreatectomy and splenectomy was performed. The patient was discharged home on day 8 post-surgery. During the follow-up in the outpatient clinic, 2 months after operation, the patient was doing well with no evidence of diabetes mellitus.

## DISCUSSION

We reported a case of an adult patient with a transected pancreas, initially treated with preservation of the distal pancreas. Post-operatively, the patient developed persistent low-output pancreatic fistula and was treated with near-total pancreatectomy.

The main concern with extensive pancreatic resection is endocrine insufficiency. Incidence of diabetes mellitus post-pancreatectomy is variable according to the extent of resection and prior chronic pancreatitis, ranging from 8 to 50% [7]. Therefore, internal drainage procedures such as pancreatico-jejunstomy were performed, which are now unfavorable due to its frequent failure [7, 8]. Transpapillary endoscopic stenting and drainage via ERCP has also been described [8, 9]. Wen et al. reported a small series of 12 patients undergoing bridge stenting internal drainage of the severed main pancreatic duct. In such cases, stents were placed across the injured duct either surgically or via endoscope. The short term 5-month follow up showed similar complications rate compared to surgery except for endocrine insufficiency that occurred in only one patient [9].

In our patient with an isolated grade IV injury, given the viability of the gland, we opted for conservative approach by ligating the main duct plus wide drainage to preserve pancreatic tissue and function in such young patient. However, he developed a persistent low-output pancreatic fistula requiring distal pancreatectomy. Pancreatic fistula was reported as the most common post-operative pancreatic complication ranging from 11 to 37% if there is ductal involvement [8]. Conservative management should be expected to consume more resources and time. It also requires active participation of the patient and home care. Within a 2-month follow-up, the patient did not show evidence of endocrine or exocrine pancreatic insufficiency; however, he did not continue the follow-up. Pancreatic insufficiency following resection post-trauma is variable and depends on the extent of excision [9, 10]. A prior study on patients who underwent either distal or proximal post-trauma pancreatectomy, nearly 30% of patients who underwent a proximal pancreatectomy required insulin on discharge, no patients with distal pancreatectomy were insulin dependent and no patients in either group developed exocrine insufficiency [10]. We attempted a conservative approach in our patient which eventually failed requiring a resectional surgery. This only adds to the dilemma of the approach to such cases. Multidisciplinary team is needed including endoscopic and interventional radiology. Finally, socio-economical and patients’ factor may have an impact on the outcome.

## CONCLUSIONS

Surgical management of pancreatic transection has evolved over the decades from aggressive approaches to more conservative measures. Given the lack of large series and clinical experience, no universal consensus exists, except for applying damage control surgery and resuscitation principles in critically unstable patients. For transections of the main pancreatic duct, most recommend excision of the distal pancreas. Concerns over the iatrogenic complications of wide excisions, particularly diabetes mellitus, have led to reconsideration and more conservative approaches, but it may fail in some cases.

## CONFLICT OF INTEREST STATEMENT

None declared.

## FUNDING

This case report has no funding; however, we acknowledge the Qatar National Library for covering the open access publication fees.

## AUTHORS’ CONTRIBUTIONS

A.R., Z.B., H.J., S.R., R.P., A.E., H.A. and A.M. have substantial contribution in terms of data collection, interpretation, writing and approval of this manuscript.

## ETHICS APPROVAL AND CONSENT TO PARTICIPATE

This case report was approved by the medical research center at Hamad medical corporation, Qatar (IRB# **MRC-04-22-446).** Data were kept confidential and anonymous, and no identified images were used.

## DATA AVAILABILITY

Not applicable.

## References

[1] Ordoñez CA , Parra MW, Millán M, Caicedo Y, Padilla N, Guzmán-Rodríguez M, et al. Pancreatic damage control: the pancreas is simple don't complicate it. Colomb Med (Cali) 2020;51:e4164361. 33795904 10.25100/cm.v51i4.4361 PMC7968433

[2] Petrone P , Moral Álvarez S, González Pérez M, Ceballos Esparragón J, Marini CP. Pancreatic trauma: management and literature review. Cir Esp 2017;95:123–30. 27480036 10.1016/j.ciresp.2016.05.011

[3] Ho VP , Patel NJ, Bokhari F, Madbak FG, Hambley JE, Yon JR, et al. Management of adult pancreatic injuries: a practice management guideline from the Eastern Association for the Surgery of Trauma. J Trauma Acute Care Surg 2017;82:185–99. 27787438 10.1097/TA.0000000000001300

[4] Al-Thani H , Ramzee AF, Al-Hassani A, Strandvik G, El-Menyar A. Traumatic pancreatic injury presentation, management, and outcome: an observational retrospective study from a level 1 trauma center. Front Surg 2022;8:771121. 35155546 10.3389/fsurg.2021.771121 PMC8831377

[5] Vijay A , Abdelrahman H, El-Menyar A, Al-Thani H. Early laparoscopic approach to pancreatic injury following blunt abdominal trauma. J Surg Case Rep 2014;2014:rju129. 25477017 10.1093/jscr/rju129 PMC4255133

[6] Siboni S , Kwon E, Benjamin E, Inaba K, Demetriades D. Isolated blunt pancreatic trauma. J Trauma Acute Care Surg 2016;81:855–9. 27537506 10.1097/TA.0000000000001224

[7] Kim KJ , Jeong CY, Jeong SH, et al. Pancreatic diabetes after distal pancreatectomy: incidence rate and risk factors. Korean J Hepatobiliary Pancreat Surg 2011;15:123–7. 26421027 10.14701/kjhbps.2011.15.2.123 PMC4582551

[8] Boffard KD , Brooks AJ. Pancreatic trauma—injuries to the pancreas and pancreatic duct. Eur J Surg 2000;166:4–12. 10688209 10.1080/110241500750009627

[9] Wen XD , Liu DQ, Zhang BY, Xiao L, Yan HT, Liu WH. The bridge stenting-based internal drainage in pancreatic trauma patients with main pancreatic duct injury. Updates Surg 2020;72:717–26. 32572695 10.1007/s13304-020-00807-5

[10] Mansfield N , Inaba K, Berg R, Beale E, Benjamin E, Lam L, et al. Early pancreatic dysfunction after resection in trauma: an 18-year report from a level I trauma center. J Trauma Acute Care Surg 2017;82:528–33. 28225740 10.1097/TA.0000000000001327

